# Supplementary material for: Hidden noise in immunologic parameters might explain rapid progression in early-onset periodontitis
Source: PLoS One. 2019 Nov 1;14(11):e0224615. doi: 10.1371/journal.pone.0224615 (PMC6824576; doi:10.1371/journal.pone.0224615)
Supplement: Attachment — Submitted filename: response to reviewers.docx [file pone.0224615.s002.docx]

Dear Prof. Yilmaz,

We must thank you and the two reviewers for the in depth review process and the comments they provided. We appreciate the reviewer’s concerns, they were very to the point and helpful, and we amended the manuscript as follows:

Academic editor

We now provide clarifications in the methodologies used for reproducibility and we discuss further on the meaning of the results and their validation approaches. We also changed the wording on the results that you found overly stated.

Additional requirements

1. We ensure that your manuscript meets PLOS ONE's style requirements.
2. We amended the ethics statement as you indicated.
3. We amended the subsection category “[FOR JOURNAL STAFF USE ONLY]” for the manuscript.
4. We now provide a data set file as a supportive information file.

Reviewer 1

“*One should be careful of interpretations of this work however, as the study (although elaborate and extensive) is cross-sectional. Therefore, it is not known if any of these parameters would change in response to treatment and validated their relevance as biomarkers of disease.”*

Response: We agree with the reviewer and we now write in the Discussion (amended manuscript page 14), “One obvious limitation of the current study originates from the fact that it is cross-sectional and as of that it is unknown how parameters might change in time. The changes that might appear in the anomaly status as a result of treatment is unknown, and therefore a confounding factor in the study might be a history of previous treatment.”

*“However, as they may also appreciate, this is quite an impossible task until biologically relevant combinations of microbial/immunological/genetic biomarker packages are readily available for home use.”*

Response: We responded to reviewer’s concern by writing (amended manuscript page 15) “When biologically relevant combinations of microbial/immunological/genetic biomarker packages will be available for use in the future, overlaying artificial intelligence algorithms might warn patients to visit the periodontist…”.

Reviewer 2

1. *Methods/results: Although patient demographics have been published elsewhere, it should still be provided to give an overall picture for the readers.*

Response: We now provide a table with patient demographics (Table 1).

2. *While the research question is to discriminate between rapid and slow progressing periodontal disease (based on history), pooling localized and generalized cases might also implement variability and noise, as the authors define. Although the subject numbers might be small in localized and generalized groups, it is important to look at the data with that perspective as well.*

Response: We now provide in the new figure 4 separate distributions for localized and generalized EOP patients. We report in Results (amended manuscript page 9) that “By separating localized from generalized EOP patients we found LOF score distributions to be similar in the two categories, with the generalized EOP category showing a higher maximum value (Fig 4B).”

3. *“Entropy values on data for neutrophil chemotaxis, CD4, CD8, CD20 counts and serum IgG titer against A.a. indicated the existence of possible anomalies”. This is unclear what exactly it is referring to. Does this mean that these analyses are not suitable to discriminate between EOP and LOP, or does it mean that they explain the possible exacerbations seen during the course of the diseases? Please clarify.*

Response: We clarify now that the findings explain the possible exacerbations seen during the course of the disease. We write (amended manuscript page 9) “Entropy values indicated possible data anomalies for neutrophil chemotaxis, CD4, CD8 and CD20 counts and IgG titer against *A.a.* (ATCC29523) that might explain more regularly occurring disease exacerbations in EOP patients.”

4. *The authors indicate that LOF presented relatively high sensitivity (94%) and specificity 83% in identifying EOP, while they conclude in Discussion that the LOF approach in predicting EOP should be generalized with caution due to a relatively high false positive rate (17%). This should be indicated in the Abstract.*

Response: we now indicate that in the abstract.

5. *Since no details are given regarding the analytical methods of immunological markers and cytokines, it is difficult to compare or comment on analytical errors or differences between groups. Perhaps, the authors can give a brief information about the analytical method used and the variation coefficient observed in those analysis.*

Response: We wrote a new subsection in Materials and Methods which we title “laboratory procedures”.

6. *It is obvious that this data represents a specific genetic and epigenetic population, the authors should also discuss the applicability of this approach on other populations and data or on individual cases in the clinic.*

Response: Thank you for your comment. We now write in the Discussion (amended manuscript page 12) “It must be noted that the current study was conducted on patients with a distinct genetic/epigenetic background (Japanese) and therefore extrapolating the results further to other populations needs to be performed with caution.”

7. *There is no extensive discussion on the results with validation group. Was the approach helped to clarify the diagnosis of this group as initially defined as “suspected” EOP?*

Response: We write in the Discussion (amended manuscript page 11-12), “The validation cohort is certainly a group of patients with severe disease (stage 3), but with a mean age higher than the EOP group. We can assume that anomalies can be found for a period of time and over the years the situation might change, perhaps due to treatment interventions. The smaller number of patients in the validation cohort might have prevented anomalies to be revealed.”

Yours sincerely,

George Papantonopoulos

Corresponding author
